# Measuring concerns about the COVID-19 vaccine among Japanese internet users through search queries

**DOI:** 10.1038/s41598-022-18307-4

**Published:** 2022-09-03

**Authors:** Makoto Uehara, Sumio Fujita, Nobuyuki Shimizu, Kongmeng Liew, Shoko Wakamiya, Eiji Aramaki

**Affiliations:** 1 Nara Institute of Science and Technology (NAIST), Nara, Japan; 2 Yahoo Japan Corporation, Tokyo, Japan

**Keywords:** Information technology, Public health, Human behaviour, Infectious diseases

## Abstract

With the increasing availability of the COVID-19 vaccines, vaccination has been rapidly promoted globally as a countermeasure against the spread of COVID-19. In Japan, vaccination was first introduced in February 2021. However, the amount of concern towards vaccination differs between individuals, and topics of concern include adverse reactions and side effects. This study investigated attitudes toward vaccines or vaccination during the COVID-19 pandemic across different Japanese prefectures, using Yahoo! JAPAN search queries. We first defined a vaccine concern index (VCI) by aggregating the search counts of vaccine-related queries from Yahoo! JAPAN users before examining VCI across all Japanese prefectures, accounting for gender and age. Our results demonstrated that VCI tended to be lower in more populated areas, and VCI was higher in their 20s to 40s than older people, especially in female users. Furthermore, there was a significant positive correlation (Spearman’s Rank correlation coefficient $\rho$ = 0.60, $p < 0.001$) between VCI and prefectural vaccination rate, suggesting that web searching of adverse vaccine reactions may precede actual vaccination. This could reflect the information-seeking behavior of individuals who are accepting of vaccinations.

## Introduction

The COVID-19 pandemic has disrupted lives and livelihoods and caused widespread panic worldwide^1^. The World Health Organization (WHO) claims that mass vaccination of the world’s population is critical to ending this pandemic^2^. After the development of vaccines and subsequent regulatory approvals, vaccination programs have gained traction rapidly around the world. In Japan, vaccines from Pfizer were approved by the government on 14 February 2021 and have been deployed since 17 February 2021, followed closely by vaccines from Takeda/Moderna and AstraZeneca, which were approved on 21 May 2021 and initially deployed on 23 May 2021 and 16 August 2021 respectively^3^.

However, contrary to the WHO recommendation, vaccination rates in many developed countries, including Japan, have been largely stagnant (as of December 2021)^4^. Prior to the COVID-19 pandemic, a survey of trust in vaccines conducted in 149 countries between 2015 and 2019 found that Japan was among the countries with the lowest levels of trust^5^. In February 2021 during the COVID-19 pandemic, a survey involving 26,000 participants in Japan was conducted to examine the prevalence of COVID-19 vaccine hesitancy and associated factors^6^. The most common reason for not getting vaccinated, according to respondents, was their concerns about adverse reactions, followed by doubts about vaccine effectiveness. Considering these reasons, efforts to promote vaccination programs rely critically on people’s trust in the safety and effectiveness of these vaccines. However, conducting such large-scale surveys repeatedly are resource-intensive and impractical (both for the implementer and respondents), so an alternative, naturalistic approach to quantify populational attitudes towards vaccines reactions is required.

This study aims to quantitatively reflect the different types of vaccine-related concerns across geographical areas, age and gender, by analyzing the Japanese public’s psychological reactions toward COVID-19 vaccines using an infodemiological^7^ approach, by analyzing concerns about COVID-19 vaccines from search queries. Past research has used similar approaches, such as using search queries to determine populational concerns toward COVID-19^8^. Similarly, we developed a concern index by aggregating the search counts of vaccine-related queries by prefecture. We first defined a search query-based concern index (vaccine concern index: VCI) to measure societal concerns towards COVID-19 vaccines and investigated differences by prefecture, age, and gender. VCI was calculated from vaccine-related queries searched on Yahoo! JAPAN from August to September 2021. In the following sections, we examine the external validity of the VCI in quantifying users intent behind their search queries, by calculating correlations between the VCI and actual vaccination rates by prefecture. As vaccine boosters will soon be widely available in Japan in the near future, convincing the public on the safety and effectiveness of mix-and-match of vaccines will be increasingly necessary. We believe that by examining online information-seeking behavior through this study (finding out who is seeking what kinds of information), our research can help to guide efforts to promote public uptake of vaccine boosters in the community.

## Results

### VCI results by prefecture

**Figure 1:**
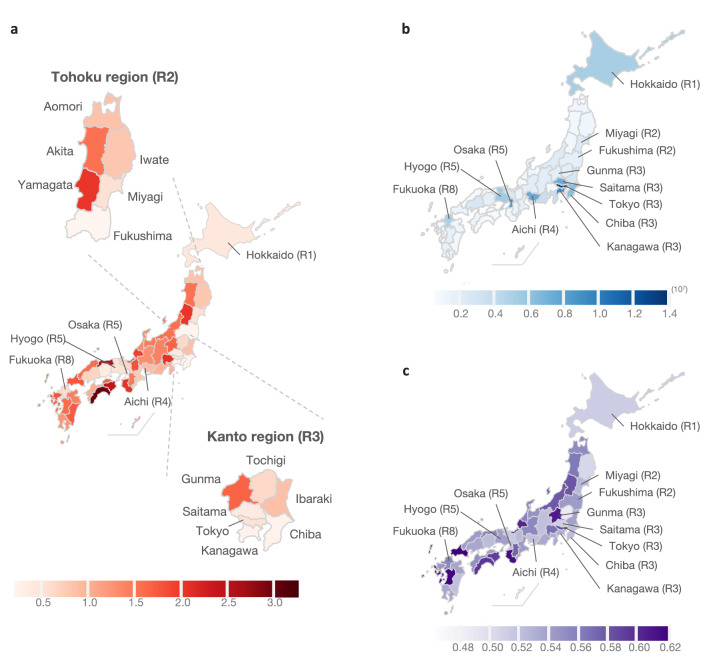
(**a**) Geographical results of the VCI in each Japanese prefecture from August to September 2021. The higher the VCI, the higher the levels of societal concern towards vaccines or vaccination. (**b**) Population of each Japanese prefecture in 2020^9^. (**c**) Vaccination rate in each Japanese prefecture (as of August 31, 2021).

Figure 1a shows the geographic results of the vaccine concern index (VCI) by geographical location from August to September 2021. Prefectures with higher VCI are shown in darker colors on the map. Table 1 shows the list of the 47 prefectures of Japan with the VCI. The results suggest that prefectures with a large population in or near each region have a low VCI (less than 1). For example, Miyagi (Tohoku (R2)), Tokyo and Kanagawa in the Kanto region (R3), Aichi in the Chubu region (R4), Osaka and Hyogo in the Kansai region (R5), Fukuoka in the Kyusyu and Okinawa region (R8). In these prefectures, the percentage of Vaccine Appointment Queries, i.e., queries that may indicate individuals’ intent to get vaccinated (e.g., “vaccine appointment” and “vaccination appointment”), from all relevant searches on vaccines (combination of Vaccine Appointment Queries and Adverse Vaccine Reactions Queries), was higher than other prefectures. This suggests that users in these regions may engage in more active web searches for vaccination appointments.

**Table 1:** List of the 47 prefectures with the VCI sorted in descending order.

Prefecture	Region	VCI	Prefecture	Region	VCI	Prefecture	Region	VCI
Kochi	R7	3.0363	Niigata	R4	1.5874	Mie	R5	0.7897
Tottori	R6	2.6072	Akita	R2	1.5608	Hiroshima	R6	0.6908
Tokushima	R7	2.4586	Nara	R5	1.5127	Kyoto	R5	0.6715
Yamanashi	R4	2.0790	Toyama	R4	1.4755	Tochigi	R3	0.6453
Wakayama	R5	2.0748	Okinawa	R8	1.4667	Fukuoka	R8	0.6275
Yamagata	R2	2.0486	Nagano	R4	1.4298	Miyagi	R2	0.5692
Fukui	R4	1.9398	Oita	R8	1.4257	Tokyo	R3	0.5567
Shiga	R5	1.8096	Ishikawa	R4	1.3611	Hyogo	R5	0.5446
Saga	R8	1.8029	Gihu	R4	1.3297	Hokkaido	R1	0.4477
Yamaguchi	R6	1.7925	Kagoshima	R8	1.2651	Saitama	R3	0.4102
Nagasaki	R8	1.7479	Ehime	R7	1.0439	Okayama	R6	0.4030
Miyazaki	R8	1.7330	Shizuoka	R4	1.0000	Osaka	R5	0.3348
Kagawa	R7	1.7123	Aichi	R4	0.9146	Chiba	R3	0.2920
Gunma	R3	1.6865	Ibaraki	R3	0.8921	Kanagawa	R3	0.2821
Kumamoto	R8	1.6241	Aomori	R2	0.8723	Fukushima	R2	0.2277
Shimane	R6	1.6028	Iwate	R2	0.8252			

The higher the VCI, the higher the levels of societal concern towards vaccines or vaccination. The Region column indicates the larger geographic region where each prefecture is located. There are eight regions in Japan: Hokkaido (R1), Tohoku (R2), Kanto (R3), Chubu (R4), Kansai (R5), Chugoku (R6), Shikoku (R7), and Kyushu and Okinawa (R8).

**Figure 2:**
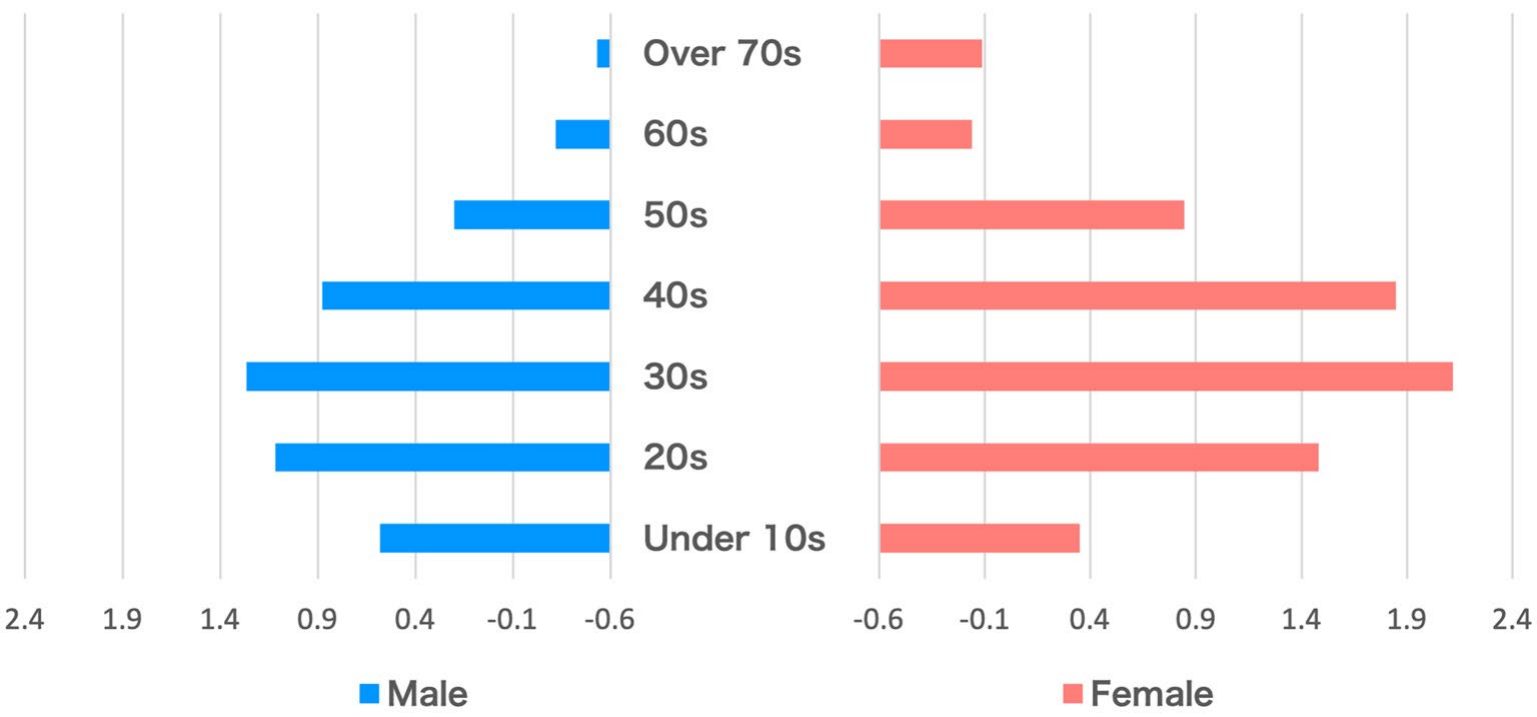
The higher the VCI, the higher the levels of societal concern towards vaccines or vaccination.

### VCI results by age and gender

Figure 2 shows the results of the VCI by age and gender from August to September 2021. Note that these are the results for Japan as a whole, not by prefecture. Generally, female users have higher VCI than male users. In particular, female users in their 20s to 40s appear to have the highest VCI among all age and gender groups. However, male users in their teens and younger had a higher VCI than female users. Common to both male and female users is that VCI is low for users over 60 years old. In this age group, the VCI is below zero, meaning that the percentage of Adverse Vaccine Reactions Queries is very small.

## Discussion

### Analysis of queries

Identifying the user’s intentions and interests through their search queries would be effective in providing more relevant results to the user^10^. We initially assumed that people who search for “vaccine appointment” or “vaccination appointment” demonstrated proactiveness in their attitudes towards vaccination. For these users, prioritizing information on vaccination venues and reservations as search results may have been facilitated through their use of internet search engines. On the other hand, we assumed that “adverse vaccine reactions” would likely be searched by people who were skeptical about vaccine. Yet, when we examined the correlation between VCI and vaccination rate^11^, as shown in Fig. 1a,c, we found a significant positive correlation between the VCI-based prefecture rank and the vaccination rate-based prefecture rank (Spearman’s $\rho$ = 0.60, $p < 0.001$): the higher the VCI, the higher the vaccination rate (as of August 31, 2021). This suggests that prefectures with higher concerns towards vaccinations and adverse reactions, were also more likely to have higher vaccination rates.

One possible interpretation of these findings could be that people who seek information about adverse reactions may be hesitant of getting vaccinated, but hesitancy does not mean that these users have decided to not get vaccinated: rather, searches for “adverse vaccine reactions” could be part of the individual’s information gathering process before deciding on accepting or avoiding vaccination. If so, perhaps providing users with appropriate information (as opposed to misinformation) about adverse reactions can help to guide these decision making processes in a constructive manner, which may eventually result in greater vaccine acceptance across the society. Such an interpretation would be consistent with psychological research on persuasion^12^, particularly through the central (elaborative) route, by providing individuals with sufficient accurate information for decision making. In this sense, the VCI might be highlighting instances of serious deliberation by people who intend to get vaccinated or who have just been vaccinated, and may be searching for adverse reactions after vaccination. Conversely, individuals opposed towards vaccination for moral or political reasons may largely be unconcerned about adverse reactions, and future research may focus on these terms as queries in understanding vaccine hesitancy.

As such, the relative placement of adverse vaccine reactions queries against vaccine appointment queries, might represent the cognitive deliberation above and beyond a baseline attitude towards vaccination, that is represented by appointment queries: an individual that might seek an appointment may search for vaccinations one or twice in booking an appointment, but an individual that is concerned and deliberating getting vaccination may be searching for adverse side effects repeatedly until reaching a satisfactory conclusion (or until the adverse reactions subside post-vaccination).

### Analysis of VCI results by prefectures

By and large, prefectures with lower VCI tended to have a relatively smaller elderly population and a larger young people population. Saitama in the Kanto region (R3), which has the sixth lowest VCI as shown in Table 1 and the fifth largest population, conducted online surveys on attitudes toward COVID-19 vaccination among young people aged 18–39 years in August 2021^13^. 70.3% of respondents answered either “vaccinated at least once”, “will definitely get vaccinated”, or “will probably get vaccinated”, suggesting that residents in that prefecture had positive attitude toward vaccination. The survey in Saitama was conducted over the same time period that our selected search queries were actually searched on the web, and we found consistent results, in that young people appear less concerned with vaccinations than the other age groups. This may help contextualize our results, as other low VCI prefectures also tend to have large populations and high percentages of young people. It could be that these younger individuals may not be as concerned about the side effects of the vaccine, and are thus less resistant towards vaccinations.

On the other hand, Fukushima in the Tohoku region (R2) has the lowest VCI even though it has a smaller population than other prefectures as shown in Fig. 1a,b. In Fukushima, the number of infections recorded was high in early August 2021, and in response, priority measures to prevent the spread of the disease was declared. Priority measures to prevent the spread of the disease is legal measures issued by the government for specific areas to prevent the spread of disease before a state of emergency is declared. In more populated prefectures such as Kanagawa and Chiba, where the VCI is low, a state of emergency had already been declared. Despite not being a highly populated prefecture, the government’s declaration following the other highly populated prefectures may have influenced people’s attitudes toward COVID-19 and vaccination.

In contrast, Gunma is the only prefecture in the Kanto region (R3) with a VCI higher than 1. This indicates that the use of vaccine appointment queries is lower in Gunma than in other prefectures in the Kanto region and that vaccination is not actively searched. The Kanto region is the most populated region in Japan, with the population of four prefectures of Tokyo, Kanagawa, Saitama, and Chiba accounting for 30% of the Japanese population (Fig. 1a,b). Compared to these prefectures, Gunma has a small population. However, VCI is less than 1 in Tochigi, which has a similar population as Gunma. This suggests that there would be some trends other than the population that are unique to Gunma.

### Analysis of VCI results by age and gender

Our results show that female users have higher VCI than male users, especially those in their 20s to 40s. Okubo et al. conducted a survey to examine the proportion of COVID-19 vaccine hesitancy and factors associated with vaccine hesitancy in Japan^6^. In their survey, 26,000 people from all prefectures in Japan participated in an online survey conducted in February 2021. Results show that the proportion of vaccine hesitancy is particularly high among young people, females, and those living alone. Despite the timing difference, our results show a similar trend to the Okubo et al.’s survey, in that we found that females aged 15–39 years formed the highest proportion of users that avoided vaccination. One reasons for these results could be the spread of misinformation on the Internet that vaccination causes infertility^14,15^. The Japanese Society of Obstetrics and Gynecology recommends vaccination, but says more information needs to be collected on medium- and long-term safety^16^. Females who are exposed to this information are more likely to be cautious about receiving vaccination, which is reflected in the survey results.

The VCI is below zero for users over 60 years old, indicating the percentage of searches with adverse vaccine reactions queries was lower than in other age groups. This is unsurprising, given that adults over 65 years of age have higher rates of severe disease and mortality with COVID-19 infection^17,18^. Accordingly, vaccination for people over 65 years of age was prioritized, and vaccinations began in April 2021 in Japan. Okubo et al.’s study also found that the proportion of vaccine hesitancy was more than twice as high among younger people aged 15–39 years than among elderly people aged 65–79 years. There are several possible reasons for lower concerns towards vaccination amongst the elderly. For example, studies on the safety and efficacy of the COVID-19 vaccine reported that elderly people are less likely to have adverse reactions than younger people^19,20^. This result could be a consequence of our choice of “adverse vaccine reactions”. Alternatively, elderly users may not be accustomed to searching for terms and queries on topics that concern them.

### Cultural antecedents of vaccine concern

As a collectivistic, Confucian culture, Japanese often have an interdependent view of self^21^, meaning that one’s personal liberties are given lower priorities than actions that benefit society. In this regard, vaccination is seen as a prosocial activity ‘for the greater good’^22^, and guidelines issued by authoritative figures (e.g., medical professionals, government), on policies like vaccination, becomes a social norm that is widely followed. Accordingly, concerns over vaccine acceptance lean towards a practical nature, such as considerations over side effects and physical risk, and less of an moral or ideological stance (as in individualistic cultures like the USA). Our study is thus consistent in that VCI is able to quantify these practical considerations in Japanese individuals’ decision processes. Given that such ‘greater good’ arguments are common to collectivistic, Confucian cultures in East Asia (e.g., China, South Korea), the VCI may thus be adaptable to these comparable cultural contexts for infodemiological measurement of vaccine concern and hesitancy.

## Methods

### Materials

We utilized statistics on search queries related to vaccines or vaccination provided by Yahoo Japan Corporation^23^ to measure vaccine concerns for each area and each age and gender group in Japan.

Yahoo Japan Corporation hosts a wide variety of over 100 services, including web searches, on a well-known portal site called “Yahoo! Japan”^24^ and a popular mobile operating system application called “Yahoo! JAPAN App”^25^. These services are used among a wide range of users of all ages, genders, areas, occupations, and annual income, with little difference based on any of these categories. The number of monthly active users is about 70 million and 20 million for smartphones and PCs respectively. This covers about 83% of smartphone users and 64% of PC users in Japan. The proportion of male and female smartphone users is 52% and 48%, respectively. About 34% of users are aged 13–39 years, 47% of users are aged 40–64 years, and 19% of users are aged over 65 years^26^.

Based on the preliminary analysis, “adverse vaccine reactions” was selected as adverse vaccine reactions queries, while “vaccine appointment” and “vaccination appointment” were selected as vaccine appointment queries. We obtained the search count of each query from August 15 to September 15, 2021. Note that these search queries are not necessarily about COVID-19, but we assume that most of them are about COVID-19 vaccines because of the timing. The age composition of the actual sample who administered the search is 34% in their 10–30s, 57% in their 40–50s, and 9% in their 60s or over. Note that we did not take into account differences in life expectancy by gender in the analyses since smartphone use is higher among males than females in Yahoo! Japan.

The number of searches in each prefecture is defined by the value of the search UB in that prefecture. The search UB is a unique cookie that has been searched for a keyword. Age and gender are defined as the percentage of males and females among the unique Yahoo Japan IDs that searched for the keyword.

### Vaccine concern index (VCI)

We quantitatively represent the degree of concern for COVID-19 vaccines or vaccination by aggregating the search counts of vaccine-related queries from Yahoo! JAPAN users. To quantify the concern about vaccines, we defined a vaccine concern index (VCI) based on search queries as follows:

$$\begin{aligned} VCI = \frac{{AVRQ} \times w-{VAQ}}{{VAQ}}, \end{aligned}$$

where *AVRQ* is the sum of the occurrences of the query “adverse vaccine reactions”, and *VAQ* is the sum of the occurrences of the queries “vaccine appointment” and “vaccination appointment”. In addition, we defined *w* as a constant term for regularization. *w* is calculated for each prefecture and each age and gender group, respectively, as described in the following subsection. A higher *VCI* means the higher percentage of searches with adverse vaccine reactions queries relative to vaccine appointment queries, indicating a higher concern about vaccines or vaccination.

#### VCI for each prefecture

The search volume varies greatly by prefecture, e.g., more in urban areas such as Tokyo and less in rural areas. To take this into account and measure the VCI for prefecture, $w_{st}$ was defined so that the VCI for a standard prefecture was set to 1. In this study, Shizuoka was chosen as the standard prefecture. The reason for this is that Shizuoka is often chosen for test marketing in Japan because its standard of living is close to the national average and it is easy to control distribution and advertising^27^. The VCI for a prefecture *pref* is measured based on the VCI equation as follows.

$$\begin{gathered} VCI_{pref} = \frac{{AVRQ_{pref}} \times w_{st}-{VAQ_{pref}}}{{VAQ_{pref}}}, \end{gathered}$$

$$\begin{gathered} w_{st} = \frac{2{VAQ_{Shizuoka}}}{{AVRQ_{Shizuoka}}}, \end{gathered}$$

where $AVRQ_{pref}$ and $VAQ_{pref}$ are the sum of the occurrences for the adverse vaccine reactions queries and the one for the vaccine appointment queries used in the prefecture *pref*, respectively. $AVRQ_{Shizuoka}$ and $VAQ_{Shizuoka}$ are the sum of the occurrences for the adverse vaccine reactions queries and the one for the vaccine appointment queries searched in Shizuoka, respectively.

#### VCI for each age and gender group

To measure the VCI for age and gender group, $w_{av}$ was defined in a similar way. Unlike the case of the VCI for prefecture, we did not set a standard age and gender group and used the average values of *VAQ* and *AVRQ* instead. The VCI for an age and gender group *agend* is measured based on the VCI equation as follows.

$$\begin{gathered} VCI_{agend} = \frac{{AVRQ_{agend}} \times w_{av}-{VAQ_{agend}}}{{VAQ_{agend}}}, \end{gathered}$$

$$\begin{gathered} w_{av}= {} \frac{2\sum _{g \in G}{VAQ_{g}}/|G|}{\sum _{g \in G}{AVRQ_{g}}/|G|}, \end{gathered}$$

where $AVRQ_{agend}$ and $VAQ_{agend}$ are the sum of the occurrences for the adverse vaccine reactions queries and the one for the vaccine appointment queries searched by users of the target age and gender *agend*, respectively. *G* is a set of 14 groups of age (Under 10s, 20s, 30s, 40s, 50s, 60s, and Over 70s) and gender (male and female).

## Data Availability

Although the data that support the findings of this study are not allowed to be publicly available according to the privacy policy and data disclosure policy of Yahoo Japan Corporation and the Japanese privacy law, the corresponding author can comply with a reasonable request.
